# Cd(II) Sorption on Montmorillonite-Humic acid-Bacteria Composites

**DOI:** 10.1038/srep19499

**Published:** 2016-01-21

**Authors:** Huihui Du, Wenli Chen, Peng Cai, Xingmin Rong, Ke Dai, Caroline L. Peacock, Qiaoyun Huang

**Affiliations:** 1 State Key Laboratory of Agricultural Microbiology, Huazhong Agricultural University, Wuhan 430070, China; 2 Key Laboratory of Arable Land Conservation (Middle and Lower Reaches of Yangtze River), Ministry of Agriculture, College of Resources and Environment, Huazhong Agricultural University, Wuhan 430070, China; 3 University of Leeds, School of Earth and Environment, Leeds LS2 9JT, UK

## Abstract

Soil components (e.g., clays, bacteria and humic substances) are known to produce mineral-organic composites in natural systems. Herein, batch sorption isotherms, isothermal titration calorimetry (ITC), and Cd K-edge EXAFS spectroscopy were applied to investigate the binding characteristics of Cd on montmorillonite(Mont)-humic acid(HA)-bacteria composites. Additive sorption and non-additive Cd(II) sorption behaviour is observed for the binary Mont-bacteria and ternary Mont-HA-bacteria composite, respectively. Specifically, in the ternary composite, the coexistence of HA and bacteria inhibits Cd adsorption, suggesting a “blocking effect” between humic acid and bacterial cells. Large positive entropies (68.1 ~ 114.4 J/mol/K), and linear combination fitting of the EXAFS spectra for Cd adsorbed onto Mont-bacteria and Mont-HA-bacteria composites, demonstrate that Cd is mostly bound to bacterial surface functional groups by forming inner-sphere complexes. All our results together support the assertion that there is a degree of site masking in the ternary clay mineral-humic acid-bacteria composite. Because of this, in the ternary composite, Cd preferentially binds to the higher affinity components-i.e., the bacteria.

Soils are complex systems possessing a very high metal-binding capacity, primarily owing to various surface reactive solid particles such as minerals, bacteria and humic substances^1^,^2^,^3^,^4^,^5^,^6^. These components interact with each other to form composites that influence the chemical behaviour of heavy metals differently compared to the isolated end-member phases^7^,^8^,^9^,^10^. Understanding the speciation and distribution of trace metals in complex systems (clay minerals/bacteria/humic substances) is crucial in predicting the mobility, bioavailability and toxicity of heavy metals in natural soils and associated environments.

Clay minerals can sorb notable quantities of a variety of heavy metals in geologic systems due to their large specific surface areas, reactive surface properties and high cationic exchange capacities^11^,^12^. Previous work shows that Cd ions mainly form outer-sphere complexes on the permanently charged sites of montmorillonite^13^, and inner-sphere surface complexes on the variable charged edge sites (≡SOH) at pH > 7^14^. Bacterial cells provide a significant reactive surface for heavy metal adsorption due to the abundance of anionic functional groups of carboxyl, amine, hydroxyl, phosphoryl, and sulfhydryl^15^,^16^,^17^. Humic acid (HA), being a component of natural soil, is also capable of binding significant amounts of heavy metals as a result of the phenolic, carboxylic acid and quinone groups present in its structure^18^,^19^.

In soils and sediments, minerals are commonly found spatially associated with organic substances via hydrogen bonding, Van der Waals forces, electrostatic interaction, covalent bonding^20^,^21^. The physical and chemical properties of the mineral-organic composites differ remarkably from their single component end-members, particularly in terms of electrical double layer properties, surface charges and the types and amounts of functional groups available for metal adsorption^9^,^10^,^22^. It has been reported that mineral-organic complexes have metal adsorption properties quite different from pure minerals. For instance, Wu *et al.*^23^ find that maximum sorption of Cd on humic acid-modified Ca-montmorillonite increased by 19.2% compared to adsorption on pure clay end-member. Zhu *et al.*^24^ report that the initial rates and capacities for the sorption of Cu and Cr on goethite-*B. thuringiensis* complexes are greater than pure goethite. Generally, composites sorbents display sorption behaviour that is either additive or non-additive, i.e., the composite adsorption behaviour is either the sum of the individual end-member metal adsorptivities (the additivity rule), or it is not. To date most studies report non-additive adsorption behaviour for heavy metals in mineral-organic composite systems. There is, however, no singly adopted explanation for this behaviour. For example, Small *et al.*^25^ report that the maximum sorption of Sr^2+^ on amorphous hydrous ferric oxide (HFO)-*S. alga* composites (0.034 mmol/g) is less than the combined sorption of the two end-member components (0.041 mmol/g), suggesting non-additive adsorption, and explain this as a result of surface site masking when HFO and bacteria are associated together in a composite. In contrast, Chen *et al.*^26^ observe that the nonliving cells-goethite composite retain 82% more Zn than that predicted by their individual behaviour, which is due to that previously unexposed sites become available with certain nonliving cell-goethite combination. Similarly, Fang *et al.*^27^ show that montmorillonite-*P. putida* composites provide more reactive sites than pure montmorillonite, with the measured maximum adsorption of Cu(II) on the composites 42.5% larger than the predicted value. Recent works by Moon and Peacock^9^,^10^ suggest that heavy metal adsorption behaviour on mineral-organic composites is in fact a function of the fraction of organic material in the composite and the affinity of the heavy metal for organic binding. They show that when iron (hydr)oxide-organic composites are dominated by organic material, and adsorption involves heavy metals with high affinity for organic complexation, adsorption behaviour deviates most strongly from additivity. They attribute this to a change in the surface charge of the composites compared to the isolated end-member phases: specifically, in organic-dominated iron (hydr)oxide composites, and below the mineral point of zero charge (pH_PZC_), the positive charge of the mineral is dampened in the presence of the organic while the negative charge of the organic is dampened in the presence of the mineral. This results in enhanced cation adsorption in the mid-high pH regime where adsorption to the mineral fraction dominates, and reduced cation adsorption in the mid-low pH regime where adsorption to the organic fraction dominates, compared to the adsorption expected based on additivity^9^.

Although a number of studies investigating the adsorption behaviour of metal ions on binary mineral-organic composites have been conducted, knowledge on the adsorption characteristics of heavy metals to ternary mineral-humic acid-bacteria composite is quite limited. Therefore, the binding behavior of Cd(II) was investigated in complex systems containing montmorillonite, *Pseudomonas putida* bacterial cells and humic acid (HA) using batch sorption isotherms, isothermal titration calorimetry (ITC) and extended X-ray absorption fine-structure (EXAFS) spectroscopy. We determine the partitioning behaviour and thermodynamic binding mechanisms of Cd on end-member clay mineral, bacteria, on binary clay mineral-bacteria and clay mineral-HA composites and finally on ternary clay mineral-humic acid-bacteria composite. Our new insight into the speciation and distribution of Cd at the mineral-humic acid-bacteria interface, provides a more comprehensive understanding of the effect of component interaction on the sorption processes of heavy metals, and thus the biogeochemical cycling and ultimate fate of contaminants in natural soils and sediments.

## Results

### Adsorption of Cd

**Figure 1:**
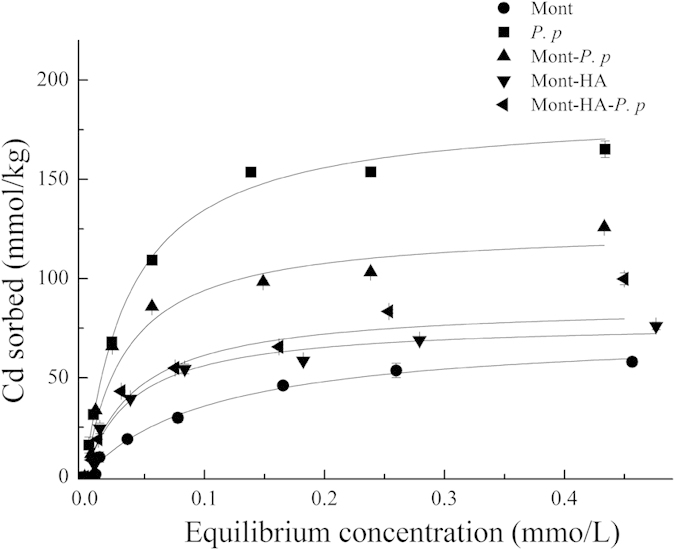
Sorption isotherms of Cd (II) on montmorillonite (Mont), bacteria (*P. p*), Mont-*P. p*, Mont-HA, and Mont-HA-*P. p* composites in the presence of 0.01 mol/L KNO_3_ at pH 5 and 28 °C.

The adsorption isotherms of Cd on the different adsorbents are presented in Fig. 1. The adsorption data conform to the Langmuir equation (*p* < 0.01, Table 1). Of all the systems studied, Mont and bacteria show the lowest and highest Cd adsorption capacities, respectively (Langmuir parameter *X*_*m*_ = 73.5 mmol Cd/kg and 185.0 mmol Cd/kg, respectively). The binary and ternary composites show Cd adsorption capacities (*X*_*m*_) that are intermediate to Mont and bacteria, ranging from 78.2 (Mont-HA) to 125.9 mmol Cd/kg (Mont-bacteria). For Mont-HA, the presence of humic acid leads to a relatively small increase in Cd adsorption capacity compared to pure Mont (from *X*_*m*_ = 73.5 mmol Cd/kg to 78.2 mmol Cd/kg, Table 1). The equilibrium constant for Cd adsorption to Mont-HA (Langmuir parameter *K*, Table 1) is significantly larger than that for Cd adsorption to Mont, indicating a higher binding affinity between Cd and the Mont-HA composite. For Mont-bacteria however, the presence of bacteria leads to a substantial increase (~70%) in Cd adsorption compared to pure Mont (from *X*_*m*_ = 73.5 mmol Cd/kg to 125.9 mmol Cd/kg, Table 1). The predicted value for Cd adsorption on Mont-bacteria, assuming additivity of the end-member adsorptivities of Mont and bacteria, is 129.2 mmol Cd/kg, which is close to the measured value. For the ternary Mont-HA-bacteria composite, the Cd adsorption capacity (*X*_*m*_) is 87.2 mmol Cd/kg; thus somewhat greater than Mont-HA, but significantly less than Mont-bacteria. Hererin, we use Mont-HA and bacteria as end-members to predict the adsorptivities of the ternary Mont-HA-bacteria composites, and we observe that the measured Cd adsorption capacity for Mont-HA-bacteria is significantly less than the predicted value (131.6 mmol Cd/kg), assuming non-additivity of the ternary Mont-HA-bacteria. Indeed, the isothermal curves for the binary & ternary composites are intermediate to the Mont and bacteria, and that the adsorption capacities increase in the composites compared to the pure Mont, suggesting that Cd is binding to both the mineral and HA and (or) bacteria components in the composites.

### Thermodynamic parameters

In the experiments to measure the thermodynamic parameters, the reaction between Cd^2+^ and each adsorbent involves two processes: (1) the dilution of Cd^2+^ and the adsorbent in the experimental solution with a continuous titration of Cd^2+^ and (2) the binding between Cd^2+^ and the adsorbent. In this study, the background calorific effect of the dilution was corrected by using the background electrolyte (0.01 mol/L KNO_3_).

**Figure 2:**
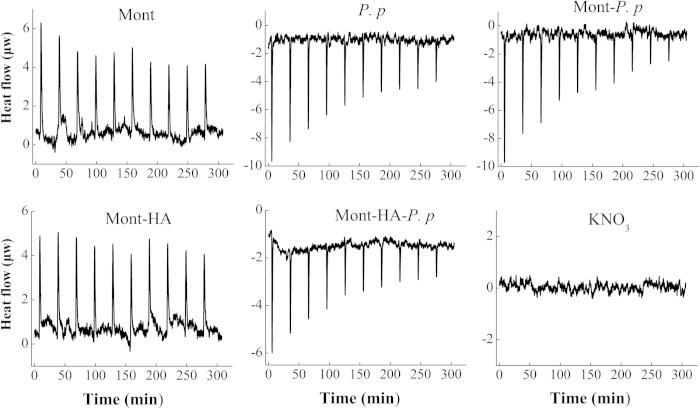
The power-time curves for Cd (II) titration to montmorillonite (Mont), bacteria (*P. p*), Mont-*P. p*, Mont-HA, and Mont-HA-*P. p* composites in the presence of 0.01 mol/L KNO_3_ at pH 5 and 28 °C.

The solid-line curves in Fig. 2 depict the raw calorimetry data for Cd adsorption on the different adsorbents. Adsorption of Cd on Mont and Mont-HA composite is an exothermic process with Δ*H* values of −10.2 kJ/mol and −8.1 kJ/mol (Table 2), respectively. In agreement with the isotherm results, the calculated thermodynamic affinity (*K*, Table 2) indicates a higher binding affinity of Cd towards Mont-HA than Mont despite a lower enthalpy for Mont-HA (−2.1 kJ/mol). For bacteria, Mont-bacteria and Mont-HA-bacteria, positive Δ*H* values are recorded after each titration. The total heat changes of the three systems follows the order: bacteria (24.9  kJ/mol) > Mont-HA-bacteria (20.2  kJ/mol) > Mont-bacteria (10.2  kJ/mol). In agreement with the isotherm results, the positive Δ*H* values for the Mont-bacteria and Mont-HA-bacteria composites suggest that the interaction between Cd and bacteria contributes to the majority of the adsorption enthalpies of Cd on these composites.

Entropy measurements can provide further information about the coordination environment of complexed metals at adsorbent surfaces, e.g., outer sphere versus inner-sphere complexation^28^. Inner-sphere complexes have positive entropies because of the displacement of water molecules from the surface adsorption sites^29^. Table 2 shows that the entropy of Cd adsorption on Mont is negative (−14.7 J/mol/K), indicative of outer-sphere adsorption. Similar outer-sphere Cd-complex on pure montmorillonite is also confirmed by EXAFS data at pH ≈ 5^13^. However, the entropy for Cd adsorption on Mont-HA is slightly positive (9.8 J/mol/K), indicative of inner-sphere adsorption. A previous study shows that Cd mainly form inner-sphere complexes on dissolved organic matter (DOM)^30^. Because HA has similar molecule structure and functional groups with DOM, the positive entropies for Cd adsorption on Mont-HA is the result of the formation of inner-sphere Cd-complexes on HA moiety. In agreement with the isotherm results, this suggests that Cd binds to both the Mont and HA component of the Mont-HA composite, and that, despite the relatively small increase in Cd adsorption capacity (*X*_*m*_, Table 1), HA does indeed provide a high affinity site for Cd binding (*K*, Tables 1 and 2), resulting in an overall entropy measurement for the Mont-HA composite that is modified from a negative to a slightly positive value.

The entropies for Cd adsorption on bacteria, Mont-bacteria and Mont-HA-bacteria are highly positive (114.4, 68.1 and 99.6 J/mol/K, respectively), indicative of inner-sphere adsorption. To date, inner-sphere adsorption of metals to different ligands such as carboxyl, phosphoryl and phenolic hydroxyl groups on bacterial cell walls^6^,^31^, and in mineral-bacteria composites^10^ has been reported in the literature. In agreement with the isotherm results, this suggests that Cd binds to both the Mont and bacteria component of the Mont-bacteria and Mont-HA-bacteria composites, and that, for Mont-bacteria, due to the relatively large increase in entropy compared to pure Mont, and for Mont-HA-bacteria, due to the relatively large increase in entropy compared to pure Mont and Mont-HA, Cd binding to the bacterial component accounts for a significant fraction of the Cd adsorbed.

Although the exact *K* values derived from the adsorption isotherms and from the calorimetry are not completely consistent with each other, they are both in the following order of bacteria > Mont-HA-bacteria > Mont-bacteria > Mont-HA > Mont, indicating a higher binding affinity for bacterial component. The negative values of the Gibbs free energy variations (−5.8 to −11.06 kJ/mol) in Table 2 confirm the feasibility of the adsorption processes and spontaneous nature of Cd adsorption on these adsorbents. By comparing |*T*Δ*S*| and |Δ*H*| values, the thermodynamic driving force of the metal-solid surface reactions can be obtained. For Mont and Mont-HA, |*T*Δ*S*| < |Δ*H*|, indicating that the adsorption reactions of Cd are enthalpically driven. For bacteria, Mont-bacteria and Mont-HA-bacteria, *T*|Δ*S*| > |Δ*H*|, indicating that the adsorption reactions are entropically driven.

### Extended X-ray absorption fine structure spectroscopy and linear combination fitting

**Figure 3:**
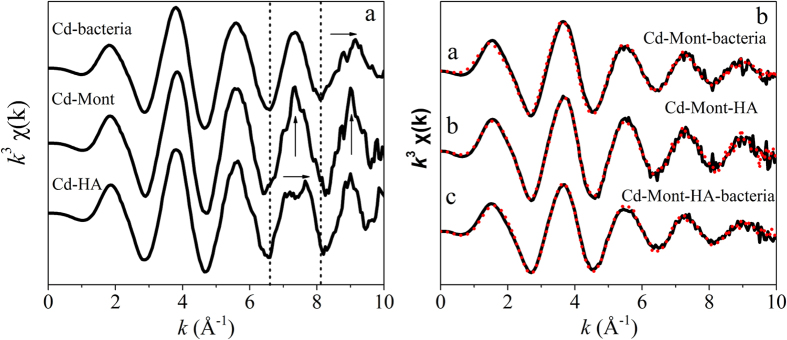
(**a**) *k*^3^-Weighted normalized Cd K-edge EXAFS spectra for montmorillonite (Mont), bacteria (*P. p*) and HA after adsorbing Cd in the presence of 0.01 mol/L KNO3 at pH 5 and 28 °C. (**b**) Normalized Cd K-edge *k*^*3*^*-*weighted EXAFS spectra of Mont-bacteria, Mont-HA and Mont-HA-bacteria composites after adsorbing Cd in the presence of 0.01 mol/L KNO_3_ at pH 5 and 28 °C. Dotted lines (red) display the best 2-component or 3-component linear combination fitting of the reference spectra. The fitting results are displayed in Table 3.

Linear combination fitting (LCF) procedure has been successfully employed to quantify the partitioning of metal adsorption on bacteria-mineral composites^9^,^22^. Hererin, we first attempt to expand this approach from binary mineral-bacteria composite to ternary mineral-HA-bacteria composite. This approach is based on the intermediate adsorption behavior of metals between the end-member component. In this study, the adsorption experiments show that Cd adsorption capacity to the ternary composites seems to be intermediate to Cd adsorption on the end members (isotherms). Moreover, the calorimetric results also suggest that Cd likely binds to both components in the ternary composite according to the intermediate entropies (99.6 J/mol/K) between Mont-HA (9.8 J/mol/K) and bacteria (114.4 J/mol/K). Consequently, it appears that we can use LCF to obtain the partitioning data of Cd in Mont-HA-bacteria systems. The model Cd K-edge (EXAFS) spectra of Mont, *P. putida* and HA after adsorbing Cd are shown in Fig. 3a. The first and second oscillations of the three spectra show no much difference, which is due to the same first coordination shell atoms, i.e., O atoms, seen eleswhere^13^,^32^,^33^. A few differences are pronounced at *k*-values of 6–10 Å^−1^. The fourth oscillation for Cd-*P. putida* and the third oscillation for Cd-HA phase shift to higher *k* values. The amplitude of the third and fourth oscillations for Cd-Mont are larger than the other two spectra. To assess the partitioning of Cd between the different components in the binary Mont-bacteria and Mont-HA composites, linear combination fitting (LCF) using the model spectra (Cd-Mont, Cd-bacteria and Cd-HA) was employed to fit each sample spectrum, using the appropriate 2-component end-member spectra. To assess the partitioning of Cd in the ternary Mont-HA-bacteria composites, linear combination fitting (LCF) using the model spectra (Cd-Mont, Cd-HA and Cd-bacteria) was employed to fit the sample spectrum, using the appropriate 3-component end-member spectra. The fitting results of the binary Mont-bacteria, Mont-HA and the ternay Mont-HA-bacteria composites are displayed as dotted lines (red) in Fig. 3b (fit parameters Table 3). The goodness of the fits is indicated by the *R*-factor (where residuals ≤0.1 indicate a good fit).

For Mont-HA, Cd adsorption to the montmorillonite component of the composite, as represented by the Cd-Mont model spectrum, accounts for the majority (~83 ± 3%, *R-*factor = 0.03) of the Cd adsorption. Although the mass ratio of HA in Mont-HA is small (~2%), a significant amount of Cd (~17 ± 3%) is bound to the HA fraction. In agreement with our previous results, this result indicates that humic acid molecules provide a high-affinity binding environment for Cd. For Mont-bacteria, Cd adsorption to the bacterial component of the composite, as represented by the Cd-bacteria model spectrum, accounts for the majority (~68 ± 2%, *R-*factor = 0.1) of the Cd adsorption. From linear additivity assumption, the percentage of Cd bound to the bacterial component should be ~71%, thus the results derived from the spectrum are quite similar to those calculated from linear additivity. For Mont-HA-bacteria, Cd adsorption to the bacterial component of the composite accounts for the majority (~76 ± 2%, *R-*factor = 0.04) of the Cd adsorption, with a lesser but significant adsorption to Mont (~21 ± 2%) and a very limited adsorption to HA (~3 ± 1%). All the fitting results suggest that the majority of Cd is associated with the bacterial component in Mont-bacteria and Mont-HA-bacteria. Specifically, in the ternary composite, more Cd is bound to bacterial surface compared to that on the binary Mont-bacteria composite.

The linear combination fitting results for the binary & ternary composites agree well with the isotherm and calorimetric results. For Mont-bacteria and, Mont-HA-bacteria, the adsorption capacity combined with the highly positive entropies demonstrate that bacteria dominate Cd adsorption on these composites. Although the mass fraction of bacteria in the binary Mont-bacteria and ternary composites do not change a lot, the bacteria-bound Cd increase from 68% in Mont-bacteria to ~76% in Mont-HA-bacteria. This result suggests that bacteria appear to prove a higher affinity binding environments for Cd in the ternary composite. All our results together support the assertion that there is a degree of site masking in the ternary composite. Because of this, in the ternary composite, Cd preferentially binds to the higher affinity components-i.e., the bacteria.

## Discussions

Our results show additive sorption behaviour of the binary clay mineral-bacteria composites for Cd, i.e., the measured maximum adsorption capacity (126.0 mmol/kg) is close to the predicted value (129.2 mmol/kg) calculated by the sum of the individual end-member metal sorptivities. Contrary to iron (hydr)oxide-bacteria composites which exhibit non-additive sorption behavior for metals^9^,^25^,^34^ due to a proportion of masking sites between the bacterial cells and the (hydr)oxide particles, it appears that no “blocking effect” occurs between the end-member of montmorillonite particles and bacterial cells in the binary composite. This result is probably due to the loose association between phyllosilicates and bacteria, since they are both negatively charged in general conditions^35^. Stronger repulsion may occur between the intact cells and montmorillonite particles due to the electrostatic energy barrier^36^. Previous work by Fang *et al.*^27^ observed an increase of the binding capacity for Cu(II) by *B. thuringiensis* or *P. putida*-montmorillonite composites which was assigned to the bridging structures of Cu(II) between both negatively charged mineral and bacterial surface. Similar bridging mechanism was proposed by Walker *et al.*^37^ for Cu^2+^ and Pb^2+^ adsorption on *Escherichia coli* cell envelopes-kaolinite composite. In this study, we did not observe this increase of sorption capacity for Cd(II) on montmorillonite-bacteria composites. The discrepancy between the current observation and those studies may result from the difference in the physiological state of the bacterial cells. In this study, the cells of *P. putida* are fresh throughout the experimental processes. In contrast, researches by Fang *et al.*^27^ and Walker *et al.*^37^ used broken or incomplete bacterial cells which had been autoclaved at 121 °C or degraded by DNase and RNase. After these treatments, bacterial cells will crack into large molecule or polymers, e.g., lipopolysaccharide, peptidoglycan, protein and phospholipids. These substances are easier than the intact bacterial cells to bind with mineral surface via a variety of mechanisms. In addition, Cd hydrolyzes less readily and the hydrated ionic radius is larger than Cu and Pb, hence is less likely to interact with the sorbent surface. Consequently, it is hard for Cd ions to bridge between the two components in the mineral-bacteria composite.

In terms of the ternary Mont-HA-bacteria, we observed non-additive sorption behaviour of the ternary composite for Cd, i.e., the measured maximum adsorption capacity (87.2 mmol/kg) is much less than the predicted value (131.6 mmol/kg), indicating a “blocking effect” in the ternary complexes. In the present study, montmorillonite is first modified by humic acid, and then the Mont-HA complex is mixed with the bacteria. Although the modification of HA does not lead to a large increase in Cd adsorption, it does provide new high affinity sites based on the *K* values (affinity) observed in isothermal adsorption and ITC results. Entropies of Cd adsorption to Mont-HA further provide evidences for the formation of these new sites. Contrast to pure Mont (−14.7 J/mol/K), entropies of the Mont-HA after adsorbing Cd is positive (9.8 J/mol/K). It is likely that the positive entropies of mineral-HA system is the result of the binding of Cd to the new sites created when the HA molecules are adsorbed to the mineral surface. Based on the sorptivities for the mineral-bacteria and mineral-HA composites, we may conclude that the blocking of surface adsorption sites on the ternary composites are mainly attributed to the interaction between bacteria and the adsorbed humic acid on mineral surface. The surface complexation reactions between humic acid and bacteria has been extensively documented, e.g., X-H(L) + R-COOH ↔ R-COOH–H(L)-X (X, R and H(L) represent humic acid, bacteria and any proton-active organic functional group, respectively)^38^,^39^. Here, in the ternary composites, the new sites on mineral-bound HA may be associated with bacterial functional groups through these surface complexation reactions, resulting in the decrease of surface reactive sites. These results suggest that HA acts as bridging between the mineral and bacterial cells. On one hand, coating of humic acid increases the reactive adsorption sites on mineral surface with less clean aluminosilicate surface. On the other hand, humic acid blocks the bacterial functional groups (vice versa) through site-specific reactions. Consequently, the surface reactive adsorption sites of the ternary composite is dependent on the intensities of these effects. Based on the above analysis, it seems that the “blocking effect” between bacteria and mineral-bound HA is more important in affecting the immobilization of Cd on the ternary composite.

Linear combination fitting of the EXAFS spectra and ITC results provide more detailed information on the distribution of Cd in the complexes due to the component interactions. For Mont-bacteria, Cd sorption to the bacterial fractions is 68%, while this percentage increases to 76% in the ternary Mont-HA-bacteria composite. These results suggest that humic acid significantly change the distribution between the mineral and bacterial fractions, i.e., promoting the combination of Cd to bacterial surface. This tendency can be verified by the thermodynamic parameters. Larger entropies for the adsorption of Cd on the ternary composites (99.6 J/mol/K) than that on the mineral-bacteria composite (68.1 J/mol/K) demonstrate the formation of more inner-sphere complexes on bacterial cell walls. In addition, higher thermodynamic affinity is observed for Cd on the ternary system (*K*, 0.2 L/mol) as compared to the binary mineral-bacteria composite (0.016 L/mol). The ternary composite displays a similar binding affinity for Cd as that of pure bacteria system (0.25 L/mol). Based on the LCF and ITC results, one may conclude that Cd is more inclined to associate with bacterial fraction due to the component interactions in the ternary composite at the studied pH 5. The work by Moon and Peacock^10^ show that Cu mobility in the binary ferrihydrite-*Bacillus subtilis* composite is controlled by sorption onto carboxyl groups of bacteria cell walls at pH < 5. Templeton *et al.*^22^ report that at least 50% of the total sorbed Pb is associated with the biofilm component in the *Buirkholderia cepacia*/Goethite composite at pH < 5.5. The vital role of bacteria in the binding of trace metals at the mineral-bacteria interfaces is highlighted. Here, our new findings indicate that the predominant role of bacterial fraction on the distribution of Cd is not changed despite the presence of HA alters the properties of the ternary composite. The above results demonstrate the universal importance of bacteria in governing the binding characteristics and distribution of heavy metals to and between multi-component interfaces containing mineral, bacteria and humic acid at mid-low pH. Despite the high mass ratio of the bacterial fractions in the composites (~50%), the preferential sorption of Cd(II) to the biofilm at low pH is mainly due to the lower p*Ka* values expected for the bacterial surface functional groups^22^. However, further studies should investigate how mass ratio changes of the components affect the immobilization and distribution of heavy metals on ternary composites.

This study still have important implications for understanding the chemical behaviors of heavy metals in particular circumstances, such as rhizosphere or bacterial clay authigenesis dominated sediments, where a much significant proportion of bacteria might be associated with phyllosilicates and/or abiotic natural orgnic matters, cadmium is selectively immobilized by bacterial communities in the complexes of various components at mid-low pH. The potential releases and activities of Cd will be reduced owing to the formation of highly stable complexes, e.g., Cd-carboxyl and Cd-sulphydryl on bacterial cell walls^40^,^41^. These pH regimes are directly relevant to acid soils, acid mine drainage environments and blackwater rivers, and it is therefore critical to measure Cd adsorption to clay mineral-humic acid-bacteria composites. This study is also fundamental for the indepth elucidation of better understanding the stabilization of organic carbon and its effects on the mobility and bioavailablity of heavy metals in soils and associated environments. The dynamic changes of organic carbon in soil aggregates due to the cell lysis, degradation of organic matter should be considered, which may have dramatic infuences on the biogeochemical cycling of trace metals.

## Materials and Methods

### Materials

Montmorillonite was purchased from Zhejiang Sanding Montmorillonite Company (Zhejiang Province, China). The clay-size fractions (<2 μm) were isolated according to the procedure of Rong *et al.*^42^. No further preparation was performed. *Pseudomonas putida* X4 strain is an aerobic gram-negative soil bacterium. The bacterial cells were cultivated in luria broth (10.0 g/L tryptone, 5.0 g/L yeast extract, 5.0 g/L NaCl) to the late-exponential growth phase as described by Wu *et al.*^43^. The fresh *P. putida* biomass was used for the adsorption experiments. Humic acid was purchased as commercially available humic acid sodium salt, supplied by Aldrich Chemical Co., and was pretreated followed the procedure of Silva *et al.*^44^. The obtained mineral, bacteria and humic acid were suspended in 0.01 mol/L KNO_3_ electrolyte.

### Preparation the composites

The Mont-bacteria mixture was reacted together at a 1:1 ratio (dry-weight basis) and shaken for 4 hours (28 °C, pH 5). The mixture was then used for the adsorption experiments without any wash procedure. The Mont-HA complex was obtained by mixing montmorillonite and HA suspension together, and shaken for 4 hours (28 °C, pH 5). The initial mass ratio of mineral to HA was 1: 0.02 (dry-weight basis). Preliminary isothermal adsorption experiment showed that the maximum adsorption of HA on montmorillonite was at 70.5 mg/g at pH 5 (see Supplementary Fig. S1), so this mass ratio enabled all the HA to associate with the mineral fractions. The mixture was then centrifuged at 8000 *g* for 12 min and washed 3 times with 0.01 mol/L KNO_3_. The supernatants from the wash procedure were subject to UV-spectrophotometry, which showed that no free HA was released from the composites (<1%). The solid phase was resuspended in 0.01 mol/L KNO_3_. The ternary Mont-HA-bacteria complex was prepared by adding bacterial suspension to the Mont-HA complex that prepared above. The initial mineral: bacteria: HA mass ratio was 1: 1: 0.02 (dry-weight basis). The mixture was shaken for 4 hours at pH 5 (28 °C), then was centrifuged at 8000 *g* for 12 min and washed 3 times with 0.01 mol/L KNO_3_. The supernatants were subject to UV-spectrophotometry, which showed that no free HA was released from the composites (<1%). The solid phase was resuspended in 0.01 mol/L KNO_3_. The final mass ratio of each fraction in the binary & ternary composites is 1: 0.02: 1 for Mont: HA: bacteria (dry-weight basis).

### Cd(II) Adsorption

All the adsorption experiments were performed at a fixed ionic strength (0.01 mol/L KNO_3_) using batch technique at room temperature (28 °C). Each sorbent (1 mL, 10 g/L, dry-weight basis) was immersed into a series of 20 mL centrifuge tubes and then different volume of Cd^2+^ solution was added. The final volume was kept at 10 mL by the addition of 0.01 mol/L KNO_3_ for each tube. The Cd concentration ranged from 0–0.5 mmol/L. The centrifuge tubes were shaken at 40 *g* for 12 h and the pH of each suspension was kept constantly at pH 5 by minor additions of 0.1 mol/L HNO_3_ or 0.1 mol/L KOH solution. Our previous adsorption kinetics experiments show that the adsorption of Cd can reach equilibrium in 12 hours. After equilibration, the final suspensions were centrifuged at 8000 *g* and filtered through 0.45 *μ*m membrane. The supernatants were acidified with HNO_3_ and the residual Cd was measured by atomic absorption spectrometry (AAS; Varin AAS240FS). The solid phase also employed Cd K-edge X-ray absorption spectroscopy (XAS) experiment.

### Isothermal Titration Calorimetry

A TAM III thermal activity monitor system (Thermometric AB, Sweden) equipped with a 1 mL stainless steel ampoule was used for the measurement. The power-time curves of Cd on montmorillonite, bacteria, mineral-HA, mineral-bacteria and mineral-HA-bacteria composites were measured at 28 °C. The sorbent suspensions (pH 5, 5 g/L, dry-weight basis) were filled into the stainless steel ampoules and placed in the calorimeter. The reaction system was stirred at 120 rev/min by a three-blade golden propeller. After a highly stable heat flow was achieved (e.g., the signal excursion <250 nw/hr), a total volume of 200 μL titrants (10 mmol/L Cd, pH 5) was injected into the reaction cell for 10 injections at a rate of 1 μL/s. An interval of 20 min followed titrant additions and the heat flow was monitored continuously. The output data were collected continuously by TAM III assistant software. Control experiment for the background heat that was not caused by Cd adsorption (i.e., the dilution heat) was conducted by titrating Cd solution (10 mmol/L, pH 5) into 0.01 mol/L KNO_3_ (pH 5). This subtraction of background heat could be found elsewhere^27^,^29^. Data processing was performed using the Origin 8.5 software on the basis of the method described by Garcia-Valls & Hatton^45^. Initially, the heat of each titration could be calculated by TAM III assistant software. Scatter diagram was plotted with titration number and the cumulative heat as the x-axis and y-axis, respectively. Then we used an equation (see Supplementary Eq. 8) to fit this scatter diagram. The relevant thermodynamic parameters, n, *K*, and Δ*H* could be determined as those values giving the best fit of this expression to the cumulative heat release curves. The Δ*G* could be calculated according to the equation: Δ*G* = −RT ln*K*, and Δ*S* was derived from Δ*G* = Δ*H* – TΔ*S.* Detailed procedures are shown in the supplementary information.

### X-ray Absorption Spectroscopy

The X-ray absorption data at the Cd K-edge (~26700 keV) were recorded at room temperature in fluorescence mode with a silicon drift fluorescence detector at beamline BL14W1 of the Shanghai Synchrotron Radiation Facility (SSRF), China. The station operates with a Si(311) double crystal monochromator. During the measurement, storage ring energy was 3.5 GeV and the beam current varied between 150–210 mA. The photon energy was calibrated with the first inflection point of the Cd K-edge in Cd metal foil. Cd-adsorbed montmorillonite, bacteria, humic acid, Mont-HA, Mont-bacteria and Mont-HA-bacteria composites samples were loaded into thin polyethylene bags, and transported immediately to the beamline for XAFS measurement. The mass percentage of Cd in the samples was ranging from 0.5~ 2%. EXAFS spectra were collected from 26511 to 27551 eV. Background subtraction and normalization of were conducted in ATHENA, a program in the IFEFFIT package^46^. To obtain information on the partitioning of Cd between the different fractions of the composites, the spectra were fitted using a linear combination fitting procedure (LCF).

## Additional Information

**How to cite this article**: Du, H. *et al.* Cd(II) Sorption on Montmorillonite-Humic acid-Bacteria Composites. *Sci. Rep.* **6**, 19499; doi: 10.1038/srep19499 (2016).

## Supplementary Material

Supplementary Information

## Figures and Tables

**Table 1:** Langmuir parameters for the adsorption of Cd to defferent systems in the presence of 0.01 mol/L KNO_3_ at pH 5 and 28 °C.

	*X*_*m*_^a^ (mmol/kg)	*K*^b^ (L/mol)	R^2^
Mont	73.5	0.0094	0.98
Bacteria	185.0	0.0267	0.99
Mont-bacteria	125.9	0.0201	0.99
Mont-HA	78.2	0.0154	0.96
Mont-HA-bacteria	87.2	0.0237	0.98

The mass ratio of each fraction in the binary and ternary composites is 1: 0.02: 1 for Mont: HA: bacteria (dry-weight basis).

^a^Maximum capacity of Cd adsorption on different components as predicted by Langmuir isotherm.

^b^Equilibrium constant related to the binding energy.

**Table 2:** Thermodynamics parameters for the adsorption of Cd on different components in the presence of 0.01 mol/L KNO_3_ at pH 5 and 28 °C.

	K (L/mol)	Δ*G* (kJ/mol)	Δ*H*(kJ/mol)	Δ*S* (J/mol/K)	R^2^
Mont	0.010	−5.8	−10.2	−14.7	0.995
Bacteria	0.025	−9.2	24.9	114.4	0.987
Mont-bacteria	0.016	−10.3	10.2	68.1	0.962
Mont-HA	0.012	−11.06	−8.1	9.8	0.952
Mont-HA-bacteria	0.020	−9.8	20.2	99.6	0.974

**Table 3:** Summary of the percentage contribution of different components for Cd binding in the binary Mont-bacteria, Mont-HA and ternary Mont-HA-bacteria composites using best fit linear combination analysis for the different composites (chi (*k*) = 2.3–10 Å^−1^).

Composites	End-member for LCF				
	Mont %	HA %	Bacteria %	Total %	*R*-factor
Mont-bacteria	32 (2.1)		68 (2.1)	100	0.1
Mont-HA	83 (2.8)	17 (2.8)		100	0.03
Mont-HA-bacteria	21 (2.3)	3 (1)	76 (2.3)	100	0.04
