# Supplementary material for: Cd(II) Sorption on Montmorillonite-Humic acid-Bacteria Composites
Source: Sci Rep. 2016 Jan 21;6:19499. doi: 10.1038/srep19499 (PMC4726142; doi:10.1038/srep19499)
Supplement: Supplementary Information [file srep19499-s1.pdf]

Supplementary Information

Cd(II) Sorption on Montmorillonite-Humic acid-Bacteria Composites

Huihui Du[1,2], Wenli Chen[1,*], Peng Cai[1,2], Xingmin Rong[1,2], Ke Dai[1,2], Caroline L. Peacock[3], Qiaoyun Huang[1,2,*]

[1]State Key Laboratory of Agricultural Microbiology, Huazhong Agricultural University, Wuhan 430070, China

[2]Key Laboratory of Arable Land Conservation (Middle and Lower Reaches of Yangtze River), Ministry of Agriculture, College of Resources and Environment, Huazhong Agricultural University, Wuhan 430070, China

[3]University of Leeds, School of Earth and Environment, LS2 9JT, UK

*Corresponding Author: phone: +86 27 87671033; fax: +86 27 87280670; email: wlchen@mail.hzau.edu.cn; qyhuang@mail.hzau.edu.cn

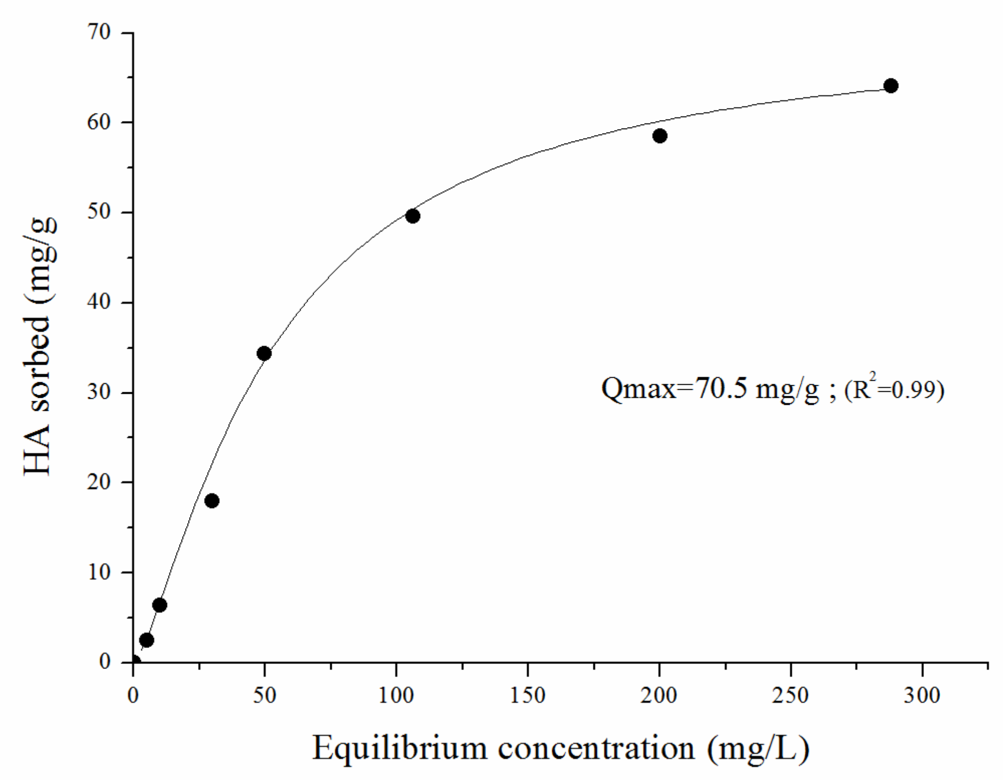

Figure S1. Equilibrium adsorption of humic acid on montmorillonite in the presence of 0.01 mol L^{-1} KNO_3 at pH 5 and 28 ℃.

ITC data analysis

The ITC data analysis was conducted using Origin 8.5 software based on the methods described by Garcia-Valls & Hatton[45]. In the present study, the heat is related to the interaction enthalpy (ΔH) by

$$\Delta Q = \Delta H\ (\Delta n_M)\ V_{\mathrm{cell}} \qquad (1)$$

Where Δn_M (mmol g^{-1}) is the changes of the total Cd bound to different components following the given injections. V_{cell} denotes the volume of the solution being titrated. The total heat released after a given number, t, of injection can be obtained by summing all the ΔQ value to give

$$Q_{\mathrm{b}} = \sum_{\mathrm{j}=1}^{\mathrm{t}} \Delta Q_{\mathrm{j}} = \Delta H\ [\mathrm{M}]_{\mathrm{b}}\ V_{\mathrm{cell}} \qquad (2)$$

where $[M]_b$ (mmol g^{-1}) represents the total adsorbed Cd after t injection. Since the sorbents may bind more than one Cd ions, the following reaction stoichiometry is used to account for Cd adsorption

$M + L \leftrightarrow ML......k_1 = [ML] / [M] [L]$

$M + ML \leftrightarrow M_2L......k_2 = [M_2L] / [M]^2 [L]$

......

$$M + M_{n-1}L \leftrightarrow M_{n-1}L......k_n = [M_nL] / [M]^n [L] \quad (3)$$

where n denotes the total number of binding sites on different components and the equilibrium constant is conditional at each pH value, then we can write

$$k_i = \frac{[M_iL]}{[M]^n [L]} = K^i \quad (4)$$

where K^i (L $mmol^{-1}$) is the global constant (β_i). Then, after some algebraic manipulation, the average fraction of Cd binding sites occupied can be shown

$$\Phi = \frac{\sum_{i=1}^{n} i[M_iL]}{\sum_{i=0}^{n}[M_iL]} = \frac{\sum_{i=1}^{n} ik_i[M]^i[L]}{\sum_{i=0}^{n} k_i[M]^i[L]} = \frac{\sum_{i=1}^{n} iK_i[M]^i}{\sum_{i=0}^{n} K_i[M]^i} = \frac{nK[M]}{1+K[M]} \quad (5)$$

The total Cd concentration is

$$[M]_T = [M] + [M]_b = [M] + [L]_T\Phi = [M] + [L]_T \frac{nK[M]}{1+K[M]} \quad (6)$$

This equation is a quadratic in [M], which can be solved to yield the free concentration of Cd in solution as a function of the total Cd and different components concentrations. The concentration of bound metal ions can then be determined to be

$$[M]_b = [M]_T - [M] = [M]_T + \frac{(1 + nK[L]_T - K[M]_T) - \sqrt{(1 + nK[L]_T - K[M]_T)^2 + 4K[M]_T}}{2K} \quad (7)$$

Therefore, the total heat released or adsorbed when the sorbents have been titrated with Cd solution when the total sorbent and Cd concentration are $[L]_T$ and $[M]_T$, respectively, is

$$Q_b = \Delta H\left([M]_T + \frac{\left(1 + nK[L]_T - K[M]_T\right) - \sqrt{\left(1 + nK[L]_T - K[M]_T\right)^2 + 4K[M]_T}}{2K}\right)V_{cell} \quad (8)$$

The relevant thermodynamic parameters, n, K, and Δ*H* can then be determined as those values giving the best fit of this expression to the cumulative heat release curves. The Δ*G* can be calculated by: Δ*G* = -RT lnK, where T is the absolute temperature (301 K), R is the universal gas constant, and K represents the equilibrium binding constant. Δ*S* is derived from Δ*G* = Δ*H* – TΔ*S*.
